# An expanded global inventory of allelic variation in the most extremely polymorphic region of *Plasmodium falciparum* merozoite surface protein 1 provided by short read sequence data

**DOI:** 10.1186/s12936-018-2475-2

**Published:** 2018-10-01

**Authors:** Harvey Aspeling-Jones, David J. Conway

**Affiliations:** Pathogen Molecular Biology Department, School of Hygiene and Tropical Medicine London, Keppel St, London, WC1E 7HT UK

**Keywords:** Polymorphism, *msp1*, Repeat sequences, Sequence mapping, De novo assembly, Molecular epidemiology, Antigen, Vaccine candidate

## Abstract

**Background:**

Within *Plasmodium falciparum* merozoite surface protein 1 (MSP1), the N-terminal block 2 region is a highly polymorphic target of naturally acquired antibody responses. The antigenic diversity is determined by complex repeat sequences as well as non-repeat sequences, grouping into three major allelic types that appear to be maintained within populations by natural selection. Within these major types, many distinct allelic sequences have been described in different studies, but the extent and significance of the diversity remains unresolved.

**Methods:**

To survey the diversity more extensively, block 2 allelic sequences in the *msp1* gene were characterized in 2400 *P. falciparum* infection isolates with whole genome short read sequence data available from the Pf3K project, and compared with the data from previous studies.

**Results:**

Mapping the short read sequence data in the 2400 isolates to a reference library of *msp1* block 2 allelic sequences yielded 3815 allele scores at the level of major allelic family types, with 46% of isolates containing two or more of these major types. Overall frequencies were similar to those previously reported in other samples with different methods, the *K1*-*like* allelic type being most common in Africa, *MAD20*-*like* most common in Southeast Asia, and *RO33*-*like* being the third most abundant type in each continent. The rare MR type, formed by recombination between *MAD20*-*like* and *RO33*-*like* alleles, was only seen in Africa and very rarely in the Indian subcontinent but not in Southeast Asia. A combination of mapped short read assembly approaches enabled 1522 complete *msp*1 block 2 sequences to be determined, among which there were 363 different allele sequences, of which 246 have not been described previously. In these data, the *K1*-*like msp*1 block 2 alleles are most diverse and encode 225 distinct amino acid sequences, compared with 123 different *MAD20*-*like*, 9 *RO33*-*like* and 6 MR type sequences. Within each of the major types, the different allelic sequences show highly skewed geographical distributions, with most of the more common sequences being detected in either Africa or Asia, but not in both.

**Conclusions:**

Allelic sequences of this extremely polymorphic locus have been derived from whole genome short read sequence data by mapping to a reference library followed by assembly of mapped reads. The catalogue of sequence variation has been greatly expanded, so that there are now more than 500 different *msp*1 block 2 allelic sequences described. This provides an extensive reference for molecular epidemiological genotyping and sequencing studies, and potentially for design of a multi-allelic vaccine.

**Electronic supplementary material:**

The online version of this article (10.1186/s12936-018-2475-2) contains supplementary material, which is available to authorized users.

## Background

The *Plasmodium falciparum* merozoite surface protein 1 (MSP1) is encoded by a gene of approximately five kilobases, with sequence regions that have been characterized as comprising relatively polymorphic or conserved blocks [1]. The most polymorphic region is block 2 that encodes a non-globular domain near the N-terminal of the protein [2], with a large number of allelic sequences classified into three major allelic family types. Two of the major types (*K1*-*like* and *MAD20*-*like*) contain highly polymorphic repeat sequences encoding variant arrays of tripeptide motifs which are distinct between the major allelic types, flanked at either end by type-specific non-repeat sequences. The third major allelic type (*RO33*-*like*) does not encode a repeat sequence and has only a few sites at which amino acid variants have been identified [3, 4]. Relatively rare MR type recombinant block 2 alleles, with homology at the 5′ end to *MAD20*-*like* and at the 3′ end to *RO33*-*like* alleles, have also been described in several surveys [3, 5–7].

Frequencies of the major allelic types are more similar across populations throughout Africa than is the case for other polymorphisms in the same gene, indicating that they may be selectively maintained within local populations [8]. There are a few lines of independent evidence indicating that MSP1 block 2 may be a significant target of acquired immunity, which could cause frequency-dependent selection to maintain the allele frequencies. All antibodies against MSP1 block 2 are against polymorphic epitopes, either major allele type-specific or discriminating further polymorphism within each of the major types [8–19].

Human serum antibodies against MSP1 block 2 have been reported to correlate with reduced prospective risk of malaria in some cohort studies of endemic populations [8–10]. Although such associations were not replicated in all studies [20, 21], a meta-analysis of several independent studies indicated an overall association between antibodies against the K1-like allelic type and protection from malaria [22]. Separately, two out of four *Aotus* monkeys experimentally immunized with a recombinant protein antigen based on the MAD20 allele of MSP1 block 2 did not develop high parasitaemia after challenge with a virulent parasite strain having a homologous allelic type [23]. Furthermore, human anti-MSP1 block 2 antibodies have been shown to inhibit parasite growth in culture in an allele-specific manner in the presence of monocytes [24], and rabbit antibodies have been found to inhibit merozoite invasion in an allele-specific manner in the presence of active complement [25].

The existing diversity of *msp1* block 2 alleles has been previously characterized in population samples by polymerase chain reaction (PCR) amplification followed by chain termination sequencing spanning the whole of block 2 [3, 4, 6, 26–29], or pyro-sequencing which produces reads of up to 300 base pairs (bp) that are long enough to span most individual *msp1* block 2 sequences [30]. The *msp1* block 2 genotypes can also be characterized at a lower level of resolution using allele type-specific PCR methods [31]. Although many *P. falciparum* clinical samples have been analysed for genome sequence variation by massively parallel paired-end short read Illumina sequencing [32], the *msp1* block 2 locus contains extremely polymorphic repetitive sequences that are complex to resolve and are therefore normally removed from genome wide analyses [33]. One potential approach to characterize highly polymorphic loci is computational assembly of the sequence reads, employing de Bruijn graph-based algorithms to split reads into sub-strings of length *k* and derive contiguous sequences by optimally linking the *k*-*mers* [34–37]. Another approach is to first construct a library of allelic reference sequences that represent a catalogue of known diversity, to which the short read sequences can be aligned. This can initially identify the main allelic type to which each sequence read maps, and the mapped reads can be further characterized by targeted de novo assembly or analysis of particular sequence motifs.

This study employs a combination of alignment and assembly of short sequence reads, to characterize *msp1* block 2 sequence polymorphism from a large sample of *P. falciparum* isolates. This has identified a larger number of allelic sequences than described previously, and illustrates an approach that could be applied to study particular loci that are highly polymorphic and contain repeat sequences, including other antigen genes.

## Methods

### Long read sequence data and generation of synthetic short reads for calibration

In order to build a database for the validation and benchmarking of novel methods developed for analysis of *msp1* block 2 short read sequence data, long read sequences deposited in GenBank were downloaded. GenBank was searched with the search terms: “*plasmodium falciparum* [organism] msp1”; “*plasmodium falciparum* [organism] msa1”; and “*plasmodium falciparum* [organism] gp195” on 4th December 2015 (GenBank search ignores the hyphen so “msp-1” and “msa-1” are effectively included). All 1831 sequence results were downloaded and curated for presence of a complete *msp1* block 2 sequence, found in 1007 of these. Removal of replicate sequences from the same strains resulted in a total of 964 sequences (381 K1-like, 350 MAD20-like, 202 RO33-like and 31 MR type). The list of studies and accession numbers for all sequences are given in Additional file 1, and a full list of sequences is given in Additional file 2. There are 290 different allelic sequences in this dataset (170 *K1*-*like*, 95 *MAD20*-*like*, 15 *RO33*-*like*, and 10 MR type).

To validate the effectiveness of a discovery approach based on short read data to detect the full spectrum of *msp1* block 2 sequences, synthetic short reads were first computationally extracted from all of the different *msp1* block 2 long read sequences in GenBank. The *msp1* block 2 sequence from each of the 964 sequences downloaded from GenBank was separately inserted at the appropriate position into a sequence file of the reference *P. falciparum* 3D7 *msp1* gene including 2 kb of sequence upstream of the start of the coding sequence (chr9:1,199,812-1206974) downloaded from PlasmoDB [38]. The python script ‘to_perfect_reads’, part of the package Fastaq (downloaded from https://github.com/sanger-pathogens/Fastaq) was used to create synthetic reads for each of the 964 *msp1* block 2 sequences with flanking regions from the 3D7 reference sequence. The majority of samples in the Pf3k dataset were sequenced by massively parallel paired-end short read sequencing of a library of randomly sheared genomic fragments on an Illumina HiSeq with 100 or 75 bp read lengths, following methods outlined in a previous report of data generation for some of these samples [39]. Therefore, synthetic reads were created at both 100 bp and 75 bp length with a mean insert size and standard deviation (SD) representative of the Pf3k data set (mean insert size of 250 bp with SD of 83 bp for 100 bp reads, and mean insert size of 277 bp with SD of 83 bp for 75 bp reads).

### Illumina paired-end short read sequence data

The Pf3k project (https://www.malariagen.net/projects/pf3k) has collated a global collection of whole genome sequence data for *P. falciparum* parasite isolates from multiple countries of Africa and Asia, and represents the largest publicly available focused resource on *P. falciparum* genetic data, in the form of paired-end short read Illumina sequences as noted above. The sequences have been sampled from individual infections, some of which contain more than one parasite genotype. Binary alignment/map (BAM) files and metadata were downloaded from Pf3k release version 4.0 (available at ftp://ngs.sanger.ac.uk/production/pf3k/release_4/). Of the 2518 individual infection isolate data downloaded, in the cases of 113 isolates having been processed on two sequencing runs only the one with the highest mean coverage was kept and the other discarded, and another five sequences that were derived from laboratory-adapted parasite lines were discarded. The resulting dataset contains sequence reads from 2400 isolates, from 26 endemic sites in 15 countries.

### Alignment of short reads to reference sequences

From each sample, paired-end short read sequences were first mapped to the *msp1* gene locus of the 3D7 reference genome. To capture reads from *msp1* block 2 sequences that would not map because of sequence polymorphism, read pairs with at least one mate mapping to any part of the *msp1* coding sequence or to the intergenic sequence within 2 kb upstream of the coding sequence (Pf3D7_09_v3:199812–1206974) were extracted from Pf3k BAM files with SAMtools [40]. The *msp1* block 2 sequence is near the 5′ end of the ~ 5 kb gene and almost all of the Illumina sequence library insert sizes are under 2 kb so the vast majority of mate pairs of reads with *msp1* block 2 sequence will map within this region.

A library of *msp1* block 2 long read sequences was created to be used as a reference for alignment of short read sequence data. First, all previously existing long read sequences downloaded from GenBank (Additional file 1) were grouped into the three *msp1* block 2 major allelic family types (*K1*-*like*, *MAD20*-*like*, and *RO33*-*like*; *MR* recombinant alleles were not needed for the library as these could be detected by alignment to the others as explained below). Sequences were aligned with all other sequences within each major allelic family type and the sequence closest to the consensus sequence (most similar to all sequences of that allelic family type) was then added to the library. This generated a first library containing one sequence per allelic type (three sequences in total). The synthetic short reads which had been generated from the long reads as described above were then aligned to the library using the basic BWA algorithm, which does not allow reads to be gapped [41]. The number of synthetic short reads mapping for each sequence was analysed, and the naturally occurring allelic sequences for which fewest component short reads were mapped to the library were then aligned, and the sequence closest to the consensus sequence was chosen to be added to yield a second reference library containing two sequences per allelic type (6 in total). This process was repeated iteratively until 10 libraries were generated with one to 10 sequences per major allelic family type (three to 30 sequences in total). The 10 reference sequence libraries were then tested by aligning the same sets of 100 bp and 75 bp synthetic reads generated from all of the 964 long read Genbank sequences to each library with BWA-MEM, which tolerates gaps in alignment [42]. Coverage was calculated for each allelic family type as the number of bases in reads aligned to the *msp1* block 2 reference library sequences of that allelic type divided by the total length of the *msp1* block 2 reference library sequences of that allelic type. Coverage was calculated for each sample by summing the coverage for the different allelic family types. Use of reference libraries having between two and 10 *msp1* block 2 sequences per allelic type gave similar numbers of reads mapping (Additional file 3). On the basis of this, a library size of five sequences per allelic type (15 sequences in total) was arbitrarily chosen as being in excess of what was required (sequences in this library are listed in Additional file 4).

Individual sequence reads were aligned to the reference library of 15 *msp1* block 2 sequences using BWA-MEM (version 0.7.5a-r405) [42] with default parameters. The resulting sequence alignment/map (SAM) files were sorted, indexed and compressed using Sambamba (version 0.6.0) [43]. SAMtools [40] was used to get the alignment statistics. For calling the presence of a given allelic family type within an individual sample, no minimum read coverage was set, as visual inspection showed that the mapping of individual reads was stringent and allele family type-specific under the BWA-MEM parameters used.

Data were analysed using the statistical analysis tool R [44] with additional package ggplot2 [45] for graphical functions. Sequences were aligned using MAFFT [46]. Assembly of short read sequences was performed with Velvet (version 1.2.10) [47].

## Results

### Evaluation of assembly of short reads

First, to test for an optimal *k*-*mer* length for de novo assembly, with a view to subsequent prospective use with Illumina short read data, assembly was performed with different parameters using synthetic short read data created from a panel of 964 long read sequences covering *P. falciparum msp1* block 2 accessed from GenBank (Additional file 1). Testing over a range of *k*-*mer* lengths between 31 and 99 demonstrated that a *k*-*mer* length of 81 generally gave best results for assembly of *msp1* block 2 sequences of any allelic type (Additional file 5), and resulted in correct assembly of 93.6% (902) of the 964 complete *msp1* block 2 sequences in GenBank. Despite this high proportion, and the observation that no assemblies contained errors, a bias was apparent as original allelic sequences of *msp1* block 2 that were longer than 200 bp were less likely to be assembled (Wilcoxon signed rank test p < 0.001; Additional file 6). To ascertain the effect of coverage depth on the assembly of longer allelic sequences, synthetic reads were generated with a range of total number of reads from the longest *msp1* block 2 allele sequence (270 bp for the laboratory isolate Palo Alto).

Analysis of the resulting assembled contigs showed that increasing coverage depth improved the probability of complete assembly of the *msp1* block 2 region (ρ = 0.96, p < 0.001; Additional file 7). As the coverage depth affected ability to assemble longer *msp1* block 2 allele sequences, which could lead to bias in use on samples having variable numbers of Illumina reads, it was decided to develop an approach to first capture *msp1* block 2 alleles in short read genome sequence data, by aligning to a composite reference library.

### Validation of alignment of short reads to a reference library of *msp1* block 2 sequences

To test the accuracy of calling the three major allelic family types of *msp*1 block 2 (*K1*-*like*, *MAD20*-*like* and *RO33*-*like*), artificial short reads generated from each of the previously reported long read sequences (Additional files 1, 2) were first aligned to the *msp1* block 2 reference library (Additional file 4). This yielded the correct allelic family type in all cases, and there was no significant difference in the coverage between the different types (Additional file 8), indicating that the approach gives robust allele calls from short read data for the three major allelic types. However, it cannot determine the presence of MR recombinant alleles, as these have sequence reads that map to both *MAD20*-*like* and *RO33*-*like* sequences in the reference library, and MR type sequences cannot be included in the library because reads from *MAD20*-*like* and *RO33*-*like* alleles would then map to 5′ and 3′ ends of these sequences, respectively.

Detecting the MR recombinant type alleles is achievable using the short read data with a separate approach, as all MR sequences contain a unique motif (5′-GGTGGTTCAGGTGCTACAGTACCT-3′, the MR identifier sequence) spanning the site of recombination between the *MAD20*-*like* and *RO*-*33*-*like* sequences. When synthetic reads were created from individual MR allele sequences within the GenBank long read dataset and aligned to the *msp1* block 2 reference library, for each allele the MR identifier sequence occurred within at least 9 reads that aligned to either *MAD20*-*like* or *RO33*-*like* sequences in the reference library. Thus MR alleles can be detected by the presence of this specific sequence within short reads aligning to the reference library. To resolve if an MR allele is present alone or in a mixed genotype infection alongside *MAD20*-*like* or *RO33*-*like* sequences, mapping to the 3′ and 5′ ends of these respective allelic sequences that are absent in the recombinant MR allele was performed.

### Detection of *msp1* block 2 allelic types from short read sequences

Aligning short read sequence data from the Pf3k dataset to the *msp1* block 2 reference library resulted in a total of 9.39 × 10^5^ aligned reads from 2400 infection isolates analysed, a mean of 391 reads per isolate. First, analysing the presence of each of the major allelic family types within isolates, at least one major allelic type was detected in 2385 (99.4%) of the isolates, and a total of 3815 counts of major allelic types were made, higher than the number of isolate samples due to the occurrence of mixed genotype infections. Overall, 46% of infection isolates had two or more different allelic family types detected, the MR recombinant alleles here being considered as a distinct type for the purpose of counting. There was a significantly higher percentage of mixed allelic type infections in Africa than in Asia, with 56% of infections containing two or more allelic types in Africa compared to 31% in Asia (Chisquare test, p < 0.001). The difference is even more marked when considering co-occurrence of three or more allelic family types, which was seen in 19% of infections in Africa compared to just 2% in Asia (Chi-square test, p < 0.001).

**Fig. 1:**
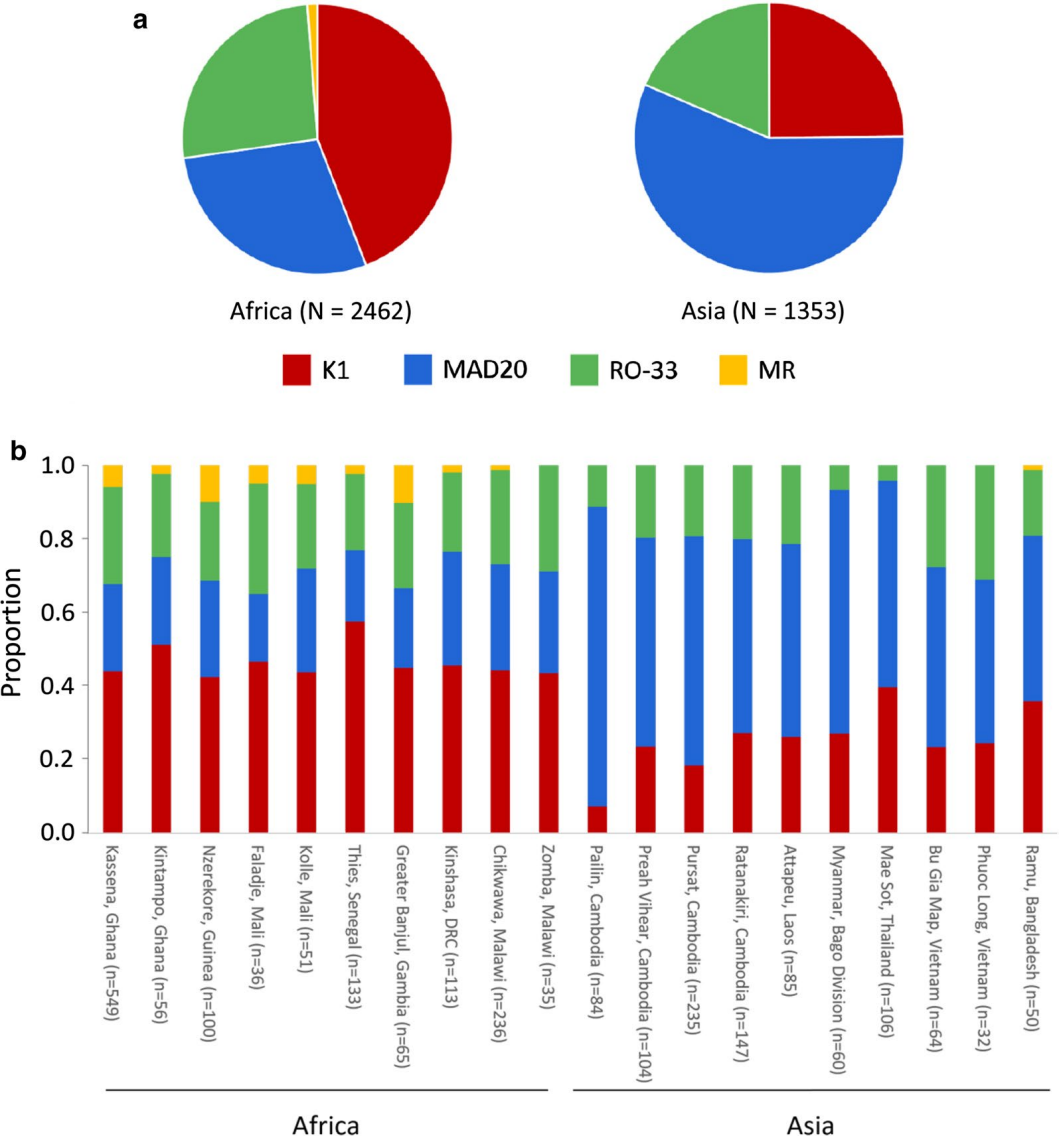
Frequencies of *msp1* block 2 major allelic types vary by geographical region. Alignment of short read data from 2400 Pf3k samples to the *msp1* block 2 reference library was used to determine the presence of *K1*- (red), *MAD20*- (blue) and *RO33*-*like* (green) *msp1* block 2 alleles in each sample, enabling a total of 3815 allele counts to be made (46% of the samples had multiple alleles detected). **a** Pie diagrams show overall frequencies in the samples from each continent. **b** The frequencies of each allelic type are shown for each sampling site where more than 30 samples were sequenced

Of all the allelic family types scored within these infection isolates, 1455 (38%) were *K1*-*like*, 1384 (36%) were *MAD20*-*like*, 860 (23%) were *RO33*-*like* and 116 (3.0%) were MR recombinants (Table 1). *K1*-*like* alleles were most common in Africa and *MAD20*-*like* alleles most common in Asia (Fig. 1, Chi-square test p < 0.001), whereas the *RO33*-*like* type did not differ in frequency between the continents. The allele frequency distributions were similar at all individual sites in Africa, contrasting with the sites in Asia (Fig. 1).

**Table 1:** Major allelic types of *msp1* block 2 determined by alignment of short reads to a library of reference sequences

Region	Number	Number (and %) of each allelic sequence type detected			
		*K1*-*like*	*MAD20*-*like*	*RO33*-*like*	*MR recombinant*
Africa	2462	1119 (45.5)	618 (25.1)	610 (24.8)	115 (4.7)
Asia	1353	336 (24.8)	766 (56.6)	250 (18.5)	1 (0.1)
All	3815	1455 (38.1)	1384 (36.3)	860 (22.5)	116 (3.0)

Short read Illumina sequence data for 2400 *P. falciparum* infection isolates in the Pf3k project were aligned to a library of reference *msp1* block 2 sequences to determine the presence of each allelic type. Overall, 3815 alleles were scored in 2385 of the isolates. MR recombinant alleles were detected by the presence of a specific MR identifier sequence, and in infections where MR recombinant alleles were detected, additional presence of *MAD20*-*like* and *RO33*-*like* alleles was checked for by searching aligned reads for the respective non-recombinant 3′ and 5′ sequences

MR recombinant alleles were detected in a total of 116 (4.8%) isolates. In 51 of these fewer than 10 reads containing the MR identifier sequence were detected and these alignments were checked by eye to confirm the presence of reads containing the MR specific sequence. The relative frequencies of MR recombinant alleles at African sites (between 2 and 10%) is similar to that seen in the small number of previous studies that tested for the MR type [3, 5, 48]. In contrast, only one Asian isolate had an MR recombinant allele detected, in Bangladesh.

### Assembly of aligned reads to determine *msp1* block 2 allelic sequences

Two separate approaches were used to extract *msp1* block 2 sequence reads for assembly, using the Velvet program [47]. One approach was to extract all reads and their mate pairs that aligned to the 3D7 reference genome sequence in the region of *msp1*. This was expected to include reads from divergent (non-*3D7*-*like*) block 2 sequences as the mate pairs of these reads would map to the more conserved *msp1* sequences flanking the polymorphic block 2 sequence. De novo assembly of reads extracted in this manner yielded complete block 2 sequence assembly for 791 (41.9%) out of the 1886 samples that had read lengths greater than 90 base pairs. The second approach was to align all reads to the *msp1* block 2 reference library and use the mapped reads for de novo assembly. This approach allowed additional sequences to be assembled, so that in total after removing identical sequences produced by the two approaches there were complete *msp1* block 2 sequences assembled from 1362 isolate samples (77.2% of all samples with read lengths greater than 90 base pairs). More than one sequence was assembled within 150 samples, with 8 samples giving three sequences and one sample giving four, overall yielding 1522 assembled sequences (Table 2 and Additional file 9).

**Table 2:** Major allelic types of *msp1* block 2 among the assembled short read sequences

Region	Number	Number (and %) of each allelic sequence type assembled			
		*K1*-*like*	*MAD20*-*like*	*RO33*-*like*	*MR recombinant*
Africa	787	367 (46.6)	185 (23.5)	204 (25.9)	31 (3.9)
Asia	735	172 (23.4)	436 (59.3)	126 (17.1)	1 (0.1)
All	1522	539 (35.4)	621 (40.8)	330 (21.7)	32 (2.1)

For short read sequences from each of the infection isolates in the Pf3k project, assembly was performed on paired end short read sequences for which one paired read mapped to a reference *msp1* gene sequence, and also on sequences that mapped to a library of reference *msp1* block 2 sequences. This yielded a total of 1522 complete *msp1* block 2 sequences from among the 2400 isolates with raw data, as some isolates did not have sufficient read depth to allow assembly of full *msp1* block 2 sequences. The number of sequences identified as belonging to each major allelic type is shown, showing similar proportions to those identified on the basis of alignment to the library of allelic reference sequences (Table 1)

Of these newly assembled allele sequences, 539 (35.3%) were *K1*-*like*, 621 (40.8%) *MAD20*-*like*, 330 (21.7%) *RO33*-*like*, and 32 (2.1%) *MR* recombinant types (Table 2). Although not all of the isolates with sequences aligned to the reference library yielded complete *msp1* block 2 allele sequence assembly, the proportions of major allelic family types in the assembled sequences (Table 2) were similar to the proportions of the corresponding allelic family types as determined by alignment to the reference library (Table 1), with *K1*-*like* alleles being most common in Africa and *MAD20*-*like* alleles most common in Asia. Analysis of the predicted translated amino acid sequences revealed 363 different block 2 alleles, of which 246 have not been described previously. Out of the 363 different block 2 alleles, the *K1*-*like* alleles are the most diverse with 225 distinct amino acid sequences, whereas there were 123 different *MAD20*-*like*, 9 *RO33*-*like* and 6 *MR* amino acid sequences. Of the 246 newly described allelic sequences, 166 were of the *K1*-*like* type, 73 were *MAD20*-*like*, five *RO33*-*like* and two were MR recombinant alleles.

### Distributions of repeat sequences within the *K1*-*like* allelic family type

**Fig. 2:**
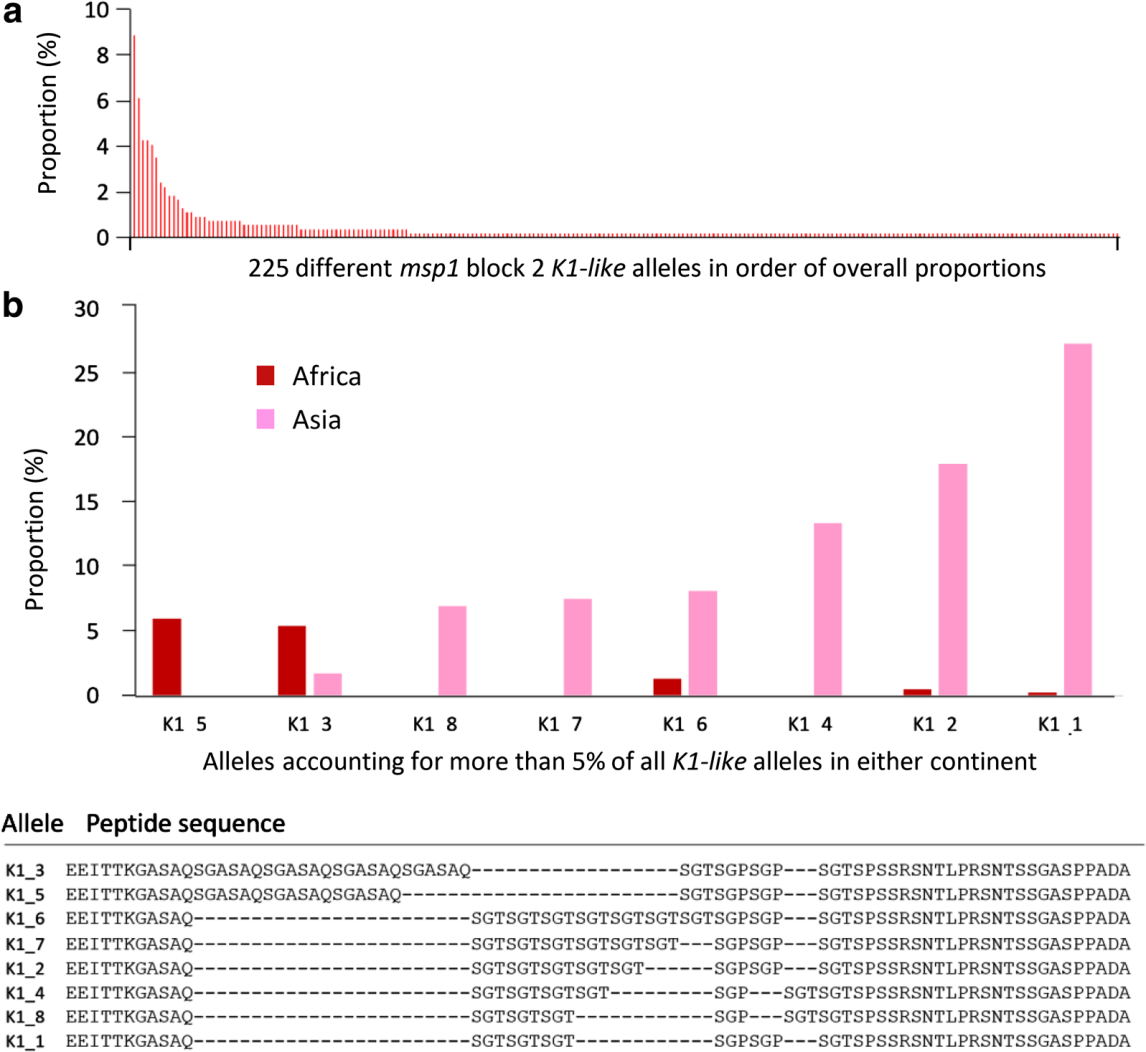
Frequency distributions of *K1*-*like* allele sequences of *msp1* block 2. **a** Overall, 225 different allele sequences were detected among 539 *K1*-*like msp1* block 2 sequences determined by assembly of short sequence reads, of which 162 occurred only once. The proportion of each as a percentage of all 539 *K1*-*like* sequences is shown. **b** Eight *K1*-*like msp1* block 2 sequences accounted for more than 5% in either Africa or Asia. The proportion of all *K1*-*like* allele sequences in Africa (367 *K1*-*like* sequences) and Asia (172 *K1*-*like* sequences), and the translated amino acid sequences of these alleles are shown. Amino acid sequences of all alleles detected are shown in Additional file 9

Within the *K1*-*like msp1* block 2 type, allelic differences are almost entirely due to variation in the repeat sequences. Due to the extremely high diversity, most of the individual *K1*-*like* sequences have been seen in only one or a few infections, but most of those that are repeatedly seen are different in Africa and Asia (Fig. 2). The African alleles tend to have longer repeat sequences (median of 12 tripeptides, range from 7 to 19) compared with Asian alleles (median of 8 tripeptides, range from five to 16) (Wilcoxon signed rank test p < 0.001). The *K1*-*like* repeats consist of four major tripeptide motifs (SAQ, SGA, SGT, and SGP), almost always beginning with the SAQ tripeptide which is only present as part of a combined motif with one of the other three tripeptides (SAQSGA, SAQSGT or SAQSGP motifs). The SGA tripeptide only occurs as part of the SAQSGA motif, whereas SGT and SGP tripeptides are commonly encoded at the end of the repeat sequence and can be part of a combined motif (for example SGTSGP) or separate motifs (for example SGTSGT or SGPSGP). In contrast with Africa where the SGA motif is present in 57.5% of all *K1*-*like* alleles sampled, only three (1.8%) of the *K1*-*like* alleles detected in Asia have an SGA motif in the tripeptide repeat, and these three are identical sequences from a single site in Bangladesh. This is consistent with previous data noting a high frequency of alleles containing the SAQSGA motif in Africa [3], but their absence in a Southeast Asian population [29], and interestingly this motif has previously been reported in a single allele from Northeast India [28], close to Bangladesh. Previously described rare *K1*-*like msp1* block 2 variants encoding SAT or SAP tripeptides [30] were not seen in any sequences.

### Distributions of repeat sequences within the *MAD20*-*like* allelic family type

**Fig. 3:**
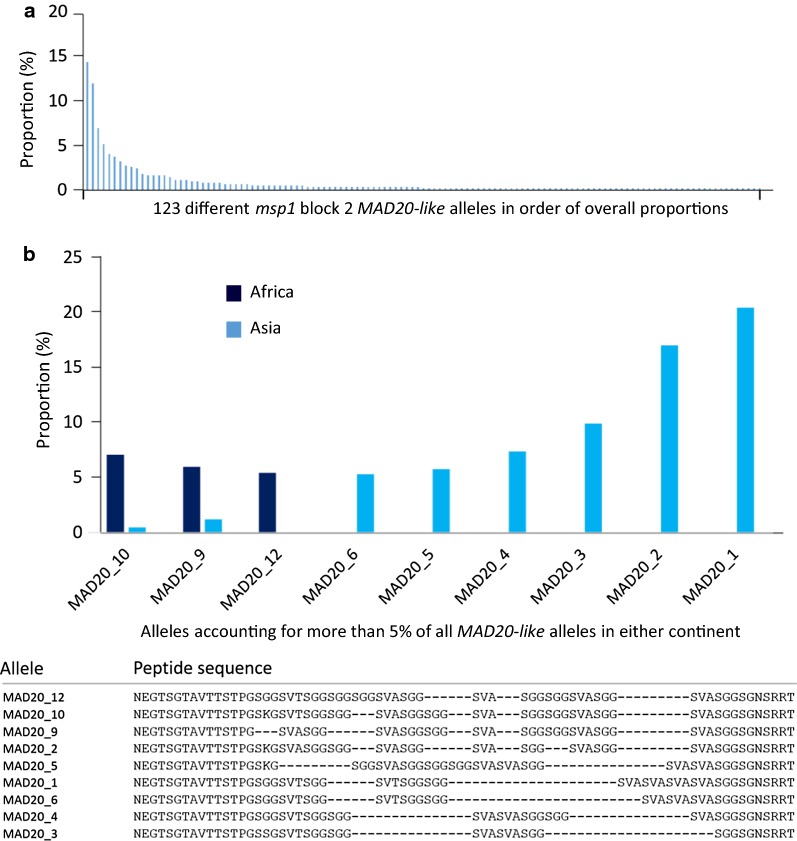
Frequency distributions of *MAD20*-*like* allele frequencies of *msp1* block 2. **a** Overall, 123 different allele sequences were detected among 621 *MAD20*-*like msp1* block 2 sequences determined by assembly of short sequence reads, of which 62 of these occurred only once. The proportion of each as a percentage of all 621 *MAD20*-*like* sequences is shown. **b** Nine *MAD20*-*like msp1* block 2 sequences accounted for more than 5% in either Africa or Asia. The proportion of these *MAD20*-*like* alleles in Africa (185 *MAD20*-*like* sequences) and Asia (436 *MAD20*-*like* sequences), and the translated amino acid sequences of these alleles are shown. Amino acid sequences of all alleles detected are shown in Additional file 9

Determining the frequency of each *MAD20*-*like* allele sequence also shows that the most common allele sequences in one continent are rare or absent in the other (Fig. 3). The *MAD20*-*like* repeats are comprised of five different common tripeptide motifs (SKG, SGG, SVA, SSG, and SVT), of which SKG can only occur as the first tripeptide and is more common in Africa (63.7% of *MAD20*-*like* alleles) than in Asia (39.4%, p < 0.001). The SKG motif is always followed by either SVA, SVT or SGG, which are all common in Asia, whereas in Africa SVT is more frequent and SGG is rare (p < 0.001). The first position in the repeat sequence can also be occupied by SGG or SVA, which can both occur multiple times in the rest of the repeat sequence. The SSG motif is almost exclusively found in Asian alleles and only occurs once, being present in 57 (13.1%) of the 436 *MAD20*-*like* Asian sequences, most of which have this as the first tripeptide followed by varying numbers of SGG and SVA repeats. Two African alleles analysed here encode a SDG tripeptide, which has only been previously reported in one allele [30].

### Sequences of *RO33*-*like* alleles differ between Africa and Asia

**Fig. 4:**
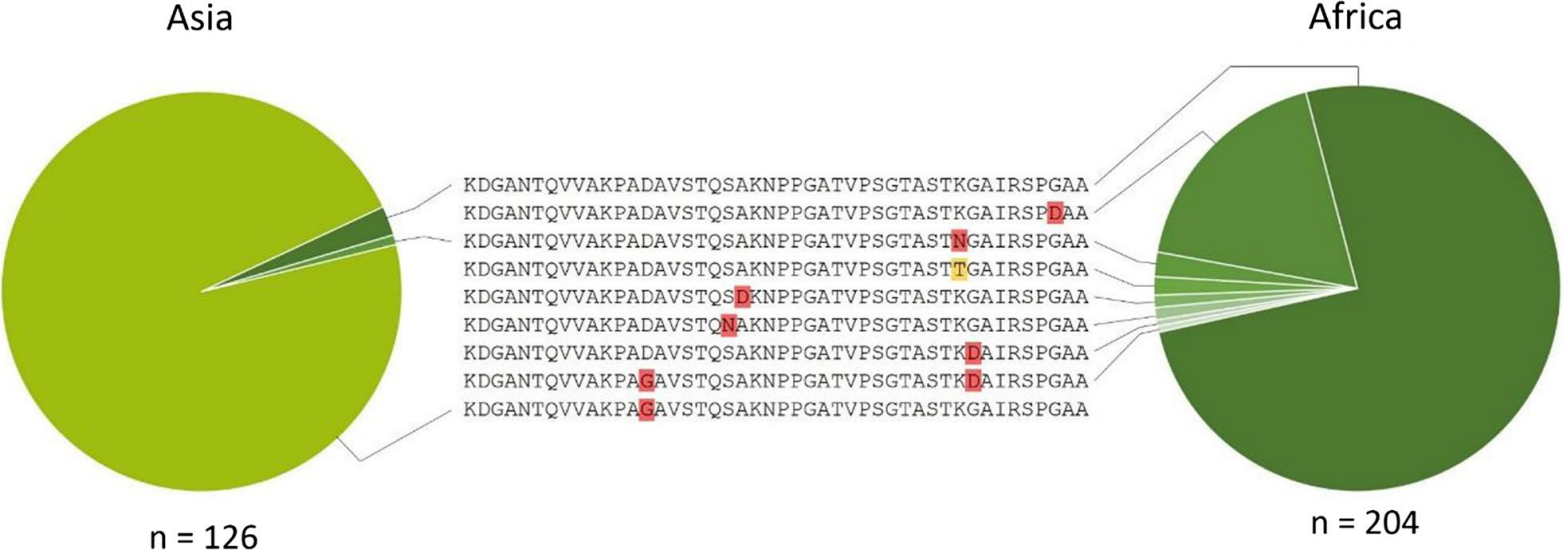
*RO33*-*like* alleles and their frequencies. Overall, 330 *RO33*-*like* alleles were determined by assembly of *msp1* block 2 short read sequences. There were six non-synonymous single nucleotide polymorphisms among these alleles, with nine different encoded amino acid sequences. Polymorphisms in the amino acid sequences are highlighted and the divergent frequencies are shown in the pie charts for Asia (left) and Africa (right)

Analysis of *RO33*-*like* sequences assembled from the short read data showed the presence of seven sub-type single nucleotide polymorphisms, six of which result in amino acid changes (Fig. 4). The exact match to the *RO33* reference allele sequence accounted for 76.0% of all *RO33*-*like* alleles sampled from Africa but only 2.3% of *RO33*-*like* alleles in Asia, consistent with previous studies based on long read sequencing [3, 4]. The next most common *RO33*-*like* allele in Africa has a single substitution (G97D), which is not seen in *RO33*-*like* alleles in Asia. Conversely, an allele with a different single substitution (D67G) accounts for 97.8% of *RO33*-*like* sequences in Asia but is not found in Africa (Fig. 4), in agreement with previous studies [4, 30]. Interestingly, the D67G substitution is seen in an African allele for the first time here, but in combination with another substitution (G91D) that is unique to Africa (Fig. 3). One previously reported allelic substitution (K90N) was detected at low frequencies in both Africa (2.0%) and Asia (0.8%), and another novel variant at this position (K90T) was seen in a single sample. Three more low frequency SNPs were identified in Africa, two of which (S73N and A74D) have not been previously reported (Fig. 4 and Additional file 9).

### MR type recombinant alleles

**Fig. 5:**
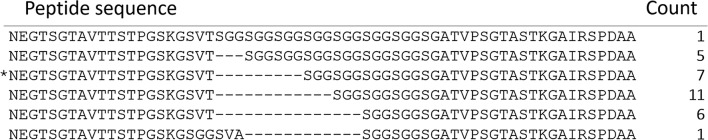
*MR* alleles and their frequencies. Six different MR type alleles, shown here, were found among the 32 *MR* recombinant allele sequences assembled. The difference between the alleles is due to expansion and contraction of the sequence encoding the serine-glycine-glycine (SGG) tripeptide, except for one allele at the bottom of the alignment that may be a rare recombinant with an *MAD20*-*like* allele having a divergent sequence. The number of times each allele was found in Africa is shown on the right. There was only one sample with an *MR* recombinant allele in Asia, which was identical to one of the African alleles (indicated with an asterisk on the left)

The 32 MR recombinant sequences that were assembled consisted of 6 different alleles. Five of these appear to have arisen from the same recombination event as they have identical 5′ *MAD20*-*like* and 3′ *RO33*-*like* sequences, and they only differ as a result of contraction or expansion of the SGG tripeptide repeat (Fig. 5). These are consistent with the range of MR recombinant alleles sequences determined previously [3, 7]. It is notable that the 3′ sequence in all MR recombinant alleles is identical to an *RO33*-*like* sequence bearing the G97D substitution which is the second most abundant *RO33*-*like* allele sequence in Africa, but absent from Asia. This suggests that the recombinant allele arose in Africa and was subsequently introduced into south Asia, where it remains rare.

A single isolate was found to contain a MR type recombinant allele with a notably different *MAD20*-*like* sequence at the 5′ end (Fig. 5), suggesting this allele arose from an independent recombination between a *MAD20*-*like* sequence and an *RO33*-*like* sequence, or recombination between an MR type allele and a different *MAD20*-*like* sequence. Previous long read sequence data also detected one MR recombinant allele with a divergent 5′ end, although this was different to the singleton sequence found here [3], suggesting that additional MR recombinant alleles may be formed although most of the MR alleles detected derive from a single recombination event.

## Discussion

The extremely polymorphic *msp1* block 2 locus in *P. falciparum* was analysed using available short-read Illumina sequences from diverse samples. Using a reference library of allelic sequences based on previous long read sequence data, it was possible to align short reads and determine the allelic family types present in almost all of the 2400 infection isolates for which data from the Pf3K project were examined. As expected, the proportion of infections containing multiple allelic family types was much higher in Africa than in Asia, and 3815 allele counts were made overall. The similarity of allele frequency distributions to those determined by long read sequencing and PCR-based genotyping of other samples indicates the validity of the approach to call *msp1* block 2 allelic family types by mapping short reads. Detecting MR recombinant alleles was possible on the basis of the unique sequence at the site of the recombination between *MAD20*-*like* and *RO33*-*like* sequences [7], and the ability to distinguish recombinant and mixed allelic types encourages the use of reference sequence libraries for genotyping complex polymorphic repeat sequences using short read data.

Targeted sequence assembly was initially attempted using paired-end sequence reads that mapped to the *msp1* locus of the 3D7 reference genome including the 5′-upstream sequence, in the hope that reads of polymorphic sequences that did not align to the reference could still be captured as pair-mates might be mapped to conserved sequences flanking block 2. However, a higher number of sequences was obtained when reads were first aligned to a library of block 2 allelic sequences prior to assembly, and the combination of both approaches here has revealed the largest dataset of *msp1* block 2 sequences to date. Of the 363 distinct allelic sequences identified among the 1522 sequences that were assembled, 246 have not been described previously. Together with 290 different sequences previously reported for this locus, more than 500 different allelic sequences have now been described for *msp1* block 2.

Among the 363 different allelic sequences identified here, there were 225 different *K1*-*like* alleles and 123 different *MAD20*-*like* alleles, the majority of which were detected only in Africa. The diversity of all allelic types was greater in Africa compared to Asia, although there was a greater diversity of different *MAD20*-*like* repeat structures in Asia, whereas many *MAD20*-*like* alleles in Africa were due to expansion of a single tripeptide-encoding repeat motif. This is in marked contrast to *K1*-*like* alleles, which in Asia almost exclusively encode a simple tripeptide repeat structure but in Africa encompass a wide range of repeat structures and lengths. It is not known to what extent the high allelic diversity within the *K1*-*like* and *MAD20*-*like* allelic types is of immunological significance. Previous analysis indicate more serological variation among the allelic sequences of the *K1*-*like* compared to the *MAD20*-*like* type [3, 9, 16], and for this reason more effort has been made to incorporate the repeat sequence variation of the *K1*-*like* alleles in recombinant antigens towards design of a future multivalent vaccine [49–51].

Targeted de novo assembly approaches can harness the increasing availability of short read genome sequence data to provide information on highly polymorphic regions and reveal rare variants. Despite the many new allelic sequences detected, it should be noted that the stringent assembly methods focused on quality, and the assembly of complete *msp1* block 2 sequences required more data than the initial mapping and detection of any sequences with particular allelic family types, so some isolates did not yield assembled sequences, and only a minority yielded more than one allelic sequence. De novo assembly is dependent on high read quality and depth, which makes it likely that many allelic sequences present at low levels in mixed infections will not be assembled. New modifications in the use of de Bruijn graphs may enhance the assembly of different alleles from mixed infections [37], or other algorithms to assemble repeat sequences from short read data might be applied [52]. Moreover, data quality in future will improve as the average length of short reads generated by Illumina or other technologies increases, so it should become possible to assemble allelic sequences at other loci that have complex polymorphisms covering longer repeats and indels, including other antigen genes which have previously been mainly surveyed by long read sequencing [53–56].

## Conclusion

The catalogue of allelic sequence variation in MSP1 block 2 of *P. falciparum* has been greatly expanded. Despite the complex repeat sequence variation and highly divergent alleles at this extremely polymorphic single locus, allelic sequences were successfully derived from whole genome short read sequence data, by mapping to a reference library followed by assembly of mapped reads. Combining with previous data, there are now more than 500 different *msp*1 block 2 allelic sequences described, providing an extensive reference for molecular epidemiological studies and potentially for design of a multi-allelic vaccine.

## Additional files

**Additional file 1.** Accession numbers of long read sequences from GenBank used to generate the *P. falciparum msp1 block 2* reference library.

**Additional file 2.** DNA sequences from GenBank used as a starting point to generate the *P. falciparum msp1 block 2* reference library.

**Additional file 3.** Effect of the number of sequences in the reference library on the number of reads mapped.

**Additional file 4.** 15 sequences of *msp1* block 2 used in the final reference library for alignment of short read sequences.

**Additional file 5.** The effect of k-mer length on the fraction of *msp1* block 2 sequences assembled by Velvet.

**Additional file 6.** Frequency distributions of length of *msp1* block 2 sequence for assembled and unassembled sequences.

**Additional file 7.** Probability of complete assembly of *msp1* block 2 is dependent on depth of coverage.

**Additional file 8.** Distribution of coverage by allelic type after alignment of dummy reads to reference library.

**Additional file 9.** Translated amino acid sequences of each of the 1522 assembled allelic sequences of *msp1* block 2.
