# Supplementary material for: An expanded global inventory of allelic variation in the most extremely polymorphic region of Plasmodium falciparum merozoite surface protein 1 provided by short read sequence data
Source: Malar J. 2018 Oct 1;17:345. doi: 10.1186/s12936-018-2475-2 (PMC6167803; doi:10.1186/s12936-018-2475-2)
Supplement: Supplementary file 1 — Additional file 1. Accession numbers of long read sequences from GenBank used to generate the P. falciparum msp1 block 2 reference library. [file 12936_2018_2475_MOESM1_ESM.pdf]

Additional file 1.

Long read sequences from GenBank used to generate the *P. falciparum msp1 block 2* reference library

Accession numbers	No. of sequences	Reference
AB116596-AB116601	6	[1][1][1][1]
AB276001-AB276018, AB300615-AB300614	20	[2]
AB502443-AB502745, AB715435-AB715519	388	[3]
AB502746-AB502795	50	[4]
AB827737-AB827762	26	[5]
AF061119-AF061151	33	[6]
AF062348-AF062349	2	[7]
AF218248	1	[8]
AF462449-AF462456, AY826427-AY826431, DQ377133-DQ377137	18	[9]
AJ635200	1	[10]
AY714585-AY714586	2	[11]
DQ026701-DQ026702	2	[12]
DQ447647	1	[13]
DQ485417-DQ485451	35	[14]
DQ855130-DQ855135	5	[15]
EU032016-EU037095	262	[16]
HM153166-HM153256	91	[17]
HQ821869-HQ821872	4	[18]
JF300128	1	[19]
JX315617, JX412318-JX412322, JX416338-JX416341	9	[20]
KP318436-KP318438	3	[21]
KR063228-KR063231	4	[22]
L10380, M77713-M77737	26	[23]
M19143-M19144	2	[24]
M32111-M32116	6	[25]
M35727/Y00087	1	[26]
M37213	1	[27]
M55001	1	[28]
X02919	1	[29]
X03371	1	[30]
X03831	1	[31]
X05624	1	[32]
X15063	1	[33]
X52962-X52963	2	[34]
X61930	1	[35]
Z35327	1	[36]

GenBank was searched with the search terms: "plasmodium falciparum [organism] msp1"; "plasmodium falciparum [organism] msa1"; and "plasmodium falciparum [organism] gp195" on 4th December 2015. All sequences containing *msp1* block2 were downloaded. Duplicate sequences from the same laboratory strain were removed leaving 964 sequences (Additional file 2). The GenBank accession numbers for the sequences are shown with the number of sequences and the reference for the study for which they were produced.

References

1. Tanabe K, Sakihama N, Kaneko A. Stable SNPs in malaria antigen genes in isolated populations. *Science* 2004, **303**:493.
2. Tanabe K, Sakihama N, Walliker D, Babiker H, Abdel-Muhsin AM, Bakote'e B*, et al.* Allelic dimorphism-associated restriction of recombination in *Plasmodium falciparum msp1*. *Gene* 2007, **397**:153-160.
3. Tanabe K, Mita T, Palacpac NM, Arisue N, Tougan T, Kawai S*, et al.* Within-population genetic diversity of *Plasmodium falciparum* vaccine candidate antigens reveals geographic distance from a Central sub-Saharan African origin. *Vaccine* 2013, **31**:1334-1339.
4. Tanabe K, Mita,T., Ranford-Cartwright,L., Palacpac,N., Sawai,H., Sakihama,N., Horibe,S., Ohmae,H., Nakamura,M., Ferreira,M.U., Escalante,A., Bjorkman,A., Farnert,A., Kaneko,A., Horii,T. Kishino,H. Geographical distribution of diversity in antigen genes and housekeeping genes of *Plasmodium falciparum*. *Unpublished* 2009.
5. Tanabe K, Zollner,G.E., Sattabongkt,J., Khuntirat,B., Honma,H., Mita,T., Tsuboi,T. and Coleman,R. Genetic diversity of *Plasmodium falciparum* in an isolated village in western Thailand. *Parasitol Int* 2015, **64**:260-266.
6. Jiang G, Daubenberger C, Huber W, Matile H, Tanner M, Pluschke G. Sequence diversity of the merozoite surface protein 1 of *Plasmodium falciparum* in clinical isolates from the Kilombero District, Tanzania. *Acta Trop* 2000, **74**:51-61.
7. Jiang G, Liu R, Daubenberger CA, Pluschke G. Sequence analysis of the MSP 1 gene of Plasmodium falciparum isolates from Hainan, China. *Zhongguo Ji Sheng Chong Xue Yu Ji Sheng Chong Bing Za Zhi* 1999, **17**:294-297.
8. Shan ZX, Yu,X.B., Li,X.R., Ma,C.L., Fang,J.M. Molecular cloning and sequence analysis of major merozoite surface antigen (gp195) gene of Plasmodium falciparum isolate FCC1/HN. *Unpublished* 1999.
9. Takala SL, Escalante AA, Branch OH, Kariuki S, Biswas S, Chaiyaroj SC, Lal AA. Genetic diversity in the Block 2 region of the merozoite surface protein 1 (MSP-1) of *Plasmodium falciparum*: additional complexity and selection and convergence in fragment size polymorphism. *Infect Genet Evol* 2006, **6**:417-424.
10. AlFadhli S, Orjih A. Sickle cells alters Plasmodium falciparum genomic DNA. *Unpublished* 2004.
11. Scopel KK, Fontes CJ, Ferreira MU, Braga EM. *Plasmodium falciparum*: IgG subclass antibody response to merozoite surface protein-1 among Amazonian gold miners, in relation to infection status and disease expression. *Exp Parasitol* 2005, **109**:124-134.
12. Joshi H, Verma A, Das MK. Malaria among Jarawas-the primitive and hostile tribe of Andaman and Nicobar Islands India. *Unpublished* 2005.
13. Colborn JM, Byrd,B.D., Koita,O.A., Krogstad,D.J. Effects of adenine/thymine content and amplicon length on estimates of starting copy number using real-time PCR with SYBR green. *Unpublished* 2006.
14. Joshi H, Valecha N, Verma A, Kaul A, Mallick PK, Shalini S*, et al.* Genetic structure of *Plasmodium falciparum* field isolates in eastern and north-eastern India. *Malar J* 2007, **6**:60.
15. Kwiek JJ, Alker AP, Wenink EC, Chaponda M, Kalilani LV, Meshnick SR. Estimating true antimalarial efficacy by heteroduplex tracking assay in patients with complex Plasmodium falciparum infections. *Antimicrob Agents Chemother* 2007, **51**:521-527.
16. Noranate N, Prugnolle F, Jouin H, Tall A, Marrama L, Sokhna C*, et al.* Population diversity and antibody selective pressure to *Plasmodium falciparum* MSP1 block2 locus in an African malaria-endemic setting. *BMC Microbiol* 2009, **9**:219.
17. Juliano JJ, Porter K, Mwapasa V, Sem R, Rogers WO, Ariey F*, et al.* Exposing malaria in-host diversity and estimating population diversity by capture-recapture using massively parallel pyrosequencing. *Proc Natl Acad Sci U S A* 2010, **107**:20138-20143.
18. Mobassir HM, Sohail,M., Ritesh,K., Oralee,B.H., Tridibes,A., Raziuddin,M. Genetic Diversity in Merozoite Surface Protein-1 and 2 among Plasmodium falciparum Isolates from Malarious Districts of Tribal Dominant State Jharkhand of India. *Unpublished* 2010.
19. Bharti PK, Shukla,M., Gautam,S.P., Sharma,Y.D., Singh,N. Genetic complexity of merozoite surface protein 1 from central India. *Unpublished* 2011.
20. Medeiros MM, Fotoran WL, dalla Martha RC, Katsuragawa TH, Pereira da Silva LH, Wunderlich G. Natural antibody response to Plasmodium falciparum merozoite antigens MSP5, MSP9 and EBA175 is associated to clinical protection in the Brazilian Amazon. *BMC Infect Dis* 2013, **13**:608.
21. Sehgal R, Kaur,H., Singh,V.P., Goyal,K., Bansal,D., Sultan,A.A. Study the molecular epidemiology and genetic diversity of malaria parasites in India and in Qatar. *Unpublished* 2014.
22. Sehgal R, Kaur,H., Bansal,D., Sultan,A.A. Genetic diversity of Plasmodium falciparum Merozoite Surface Protein 1 (block2 region) from Chandigarh and adjoining states of North India. *Unpublished* 2015.

23. Jongwutiwes S, Tanabe K, Nakazawa S, Yanagi T, Kanbara H. Sequence variation in the tripeptide repeats and T cell epitopes in P190 (MSA-1) of *Plasmodium falciparum* from field isolates. *Mol Biochem Parasitol* 1992, **51**:81-89.
24. Peterson MG, Coppel RL, McIntyre P, Langford CJ, Woodrow G, Brown GV*, et al.* Variation in the precursor to the major merozoite surface antigens of Plasmodium falciparum. *Mol Biochem Parasitol* 1988, **27**:291-301.
25. Kimura E, Mattei D, di Santi SM, Scherf A. Genetic diversity in the major merozoite surface antigen of Plasmodium falciparum: high prevalence of a third polymorphic form detected in strains derived from malaria patients. *Gene* 1990, **91**:57-62.
26. Certa U, Rotmann D, Matile H, Reber-Liske R. A naturally occurring gene encoding the major surface antigen precursor p190 of Plasmodium falciparum lacks tripeptide repeats. *EMBO J* 1987, **6**:4137-4142.
27. Chang SP, Kramer KJ, Yamaga KM, Kato A, Case SE, Siddiqui WA. Plasmodium falciparum: gene structure and hydropathy profile of the major merozoite surface antigen (gp195) of the Uganda-Palo Alto isolate. *Exp Parasitol* 1988, **67**:1-11.
28. Scherf A, Mattei D, Sarthou JL. Multiple infections and unusual distribution of block 2 of the MSA1 gene of Plasmodium falciparum detected in west African clinical isolates by polymerase chain reaction analysis. *Mol Biochem Parasitol* 1991, **44**:297-299.
29. Holder AA, Lockyer MJ, Odink KG, Sandhu JS, Riveros-Moreno V, Nicholls SC*, et al.* Primary structure of the precursor to the three major surface antigens of Plasmodium falciparum merozoites. *Nature* 1985, **317**:270-273.
30. Mackay M, Goman M, Bone N, Hyde JE, Scaife J, Certa U*, et al.* Polymorphism of the precursor for the major surface antigens of Plasmodium falciparum merozoites: studies at the genetic level. *EMBO J* 1985, **4**:3823-3829.
31. Weber JL, Leininger WM, Lyon JA. Variation in the gene encoding a major merozoite surface antigen of the human malaria parasite Plasmodium falciparum. *Nucleic Acids Res* 1986, **14**:3311-3323.
32. Tanabe K, Mackay M, Goman M, Scaife JG. Allelic dimorphism in a surface antigen gene of the malaria parasite *Plasmodium falciparum*. *Journal of Molecular Biology* 1987, **195**:273-287.
33. Myler PJ. Nucleotide and deduced amino acid sequence of the gp195 (MSA-1) gene from Plasmodium falciparum Palo Alto PLF-3/B11. *Nucleic Acids Res* 1989, **17**:5401.
34. Ranford-Cartwright LC, Balfe P, Carter R, Walliker D. Direct sequencing of enzymatically amplified DNA of alleles of the merozoite surface antigen MSA-1 gene from the malaria parasite Plasmodium falciparum. *Mol Biochem Parasitol* 1991, **46**:185-187.
35. Olafsson P, Matile H, Certa U. Plasmodium falciparum: the repetitive MSA-1 surface protein of the RO-71 isolate is recognized by mouse antibody against the nonrepetitive repeat block of RO-33. *Exp Parasitol* 1992, **74**:381-389.
36. Pan W, Tolle R, Bujard H. A direct and rapid sequencing strategy for the Plasmodium falciparum antigen gene gp190/MSA1. *Mol Biochem Parasitol* 1995, **73**:241-244.
