# Supplementary material for: An expanded global inventory of allelic variation in the most extremely polymorphic region of Plasmodium falciparum merozoite surface protein 1 provided by short read sequence data
Source: Malar J. 2018 Oct 1;17:345. doi: 10.1186/s12936-018-2475-2 (PMC6167803; doi:10.1186/s12936-018-2475-2)
Supplement: Supplementary file 3 — Additional file 3. Effect of the number of sequences in the reference library on the number of reads mapped. [file 12936_2018_2475_MOESM3_ESM.pdf]

Additional file 3.

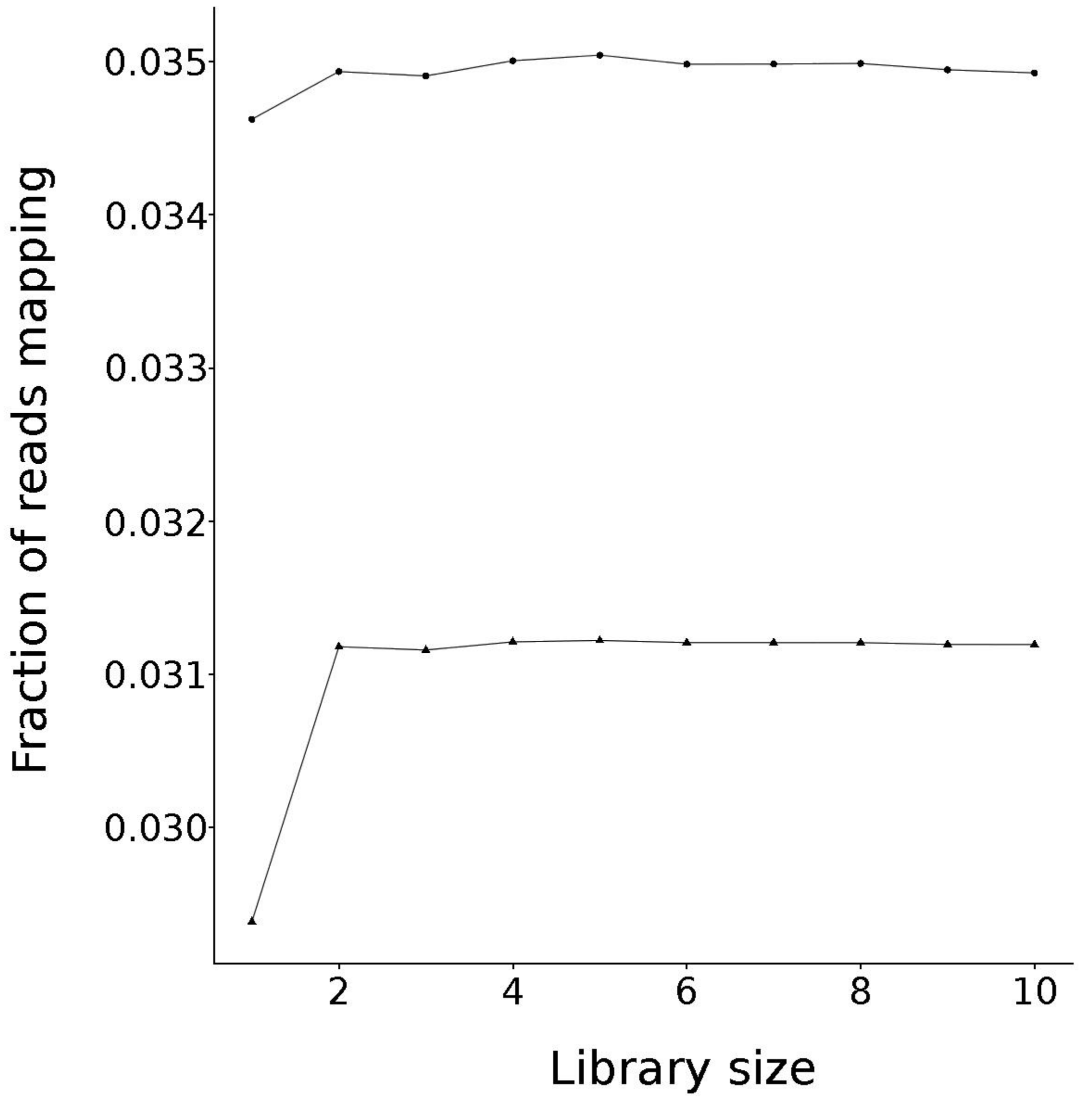

Effect of the number of sequences in the reference library on the number of reads mapped. Reference libraries were created with between one and 10 sequences per major allelic type (3 to 30 sequences total). Dummy reads created from 964 long read sequences and were aligned to the sequence libraries using BWA-MEM. The fraction of all reads that were mapped to the reference library is shown for read lengths of 100 bp (circles) and 75 bp (triangles).
