# Supplementary material for: An expanded global inventory of allelic variation in the most extremely polymorphic region of Plasmodium falciparum merozoite surface protein 1 provided by short read sequence data
Source: Malar J. 2018 Oct 1;17:345. doi: 10.1186/s12936-018-2475-2 (PMC6167803; doi:10.1186/s12936-018-2475-2)
Supplement: Supplementary file 4 — Additional file 4. 15 sequences of msp1 block 2 used in the final reference library for alignment of short read sequences. [file 12936_2018_2475_MOESM4_ESM.pdf]

Additional file 4.

15 sequences of *msp1* block 2 used in the final reference library for alignment of short read sequences. The GenBank accession numbers of all sequences used in the *msp1* block 2 reference library are grouped by allelic type. The *msp1* block 2 sequence is shown in capitals, with the block 1 and block 3 sequence fragments, also included in the library, shown in lower case.

Allelic type	Accession number	Sequence fragment used
K1	AB502525	gtattaaatgaaGAAGAAATTACTACAAAAGGTGCAAGTGCTCAAAGTGGTGCAAGTGCTCAAAGTGGTGCAAGTGCTCAAAGTGGTACAAGTGGTCCAAGTGGTCCAAGTCCATCATCTCGTTCAAACACTTTACCTCGTTCAAATACTTCATCTGGTGCAAGCCCTCCAGCTGATGCAagcgattcagat
	AB502454	gtattaaatgaaGAAGAAATTACTACAAAAGGTGCAAGTGCTCAAAGTGGTGCAAGTGCTCAAAGTGGTACAAGTGCTCAAAGTGGTACAAGTGCTCAAAGTGGTACAAGTGGTACAAGTGGTACAAGTGGTACAAGTGGTACAAGTGGTACAAGTGGTACAAGTGGTACAAGTGGTACAAGTGGTACAAGTGGTACAAGTGGTCCAAGTGGTCCAAGTGGTACAAGTCCATCATCTCGTTCAAACACTTTACCTCGTTCAAATACTTCATCTGGTGCAAGCCCTCCAGCTGATGCAagcgattcagat
	HM153224	gtattaaatgaaGAAGAAATTACTACAAAAGGTGCAAGTGCTCAAAGTGGTACAAGTGGTACAAGTCCATCATCTCGTTCAAACACTTTACCTCGTTCAAATACTTCATCTGGTGCAAGCCCTCCAGCTGATGCAagcgattcagat
	DQ485422	gtattaaatgaaGAAGAAATTACTACAAAAGGTGCGAGTGCTAGTGCTCAAAGTGGTGCAAGTGCAAGTGGTGCAAGTGCTCCAAGTGGTACAAGTGGTCCAAGTCTTCCATGTGGTACAAGTCCATCATCTCGTTCAAACACTTTACCTCGTTCAAATACTTCATCTGGTGCAAGCCCTCCAGCTGATGCAagcgattcagat
	AB502448	gtattaaatgaaGAAGAAATTACTACAAAAGGTGCAAGTGCTCAAAGTGGTACAAGTGGTACAAGTGGTCCAAGTGGTACAAGTCCATCATCTCGTTCAAACACTTTACCTCGTTCAAATACTTCATCTGGTGCAAGCCCTCCAGCTGATGCAagcgattcagat
MAD20	EU032192	gtattaaatgaaGGAACAAGTGGAACAGCTGTTACAACTAGTACACCTGGTTCAAAGGGTTCAGGTGGCTCAGTTGCTTCAGGTGGTTCAGGTGGTTCAGTTGCTTCAGTTGCTTCAGGTGGCTCAGTTGCTTCAGTTGCTTCAGGTGGCTCAGTTGCTTCAGGTGGTTCAGGTAATTCAAGACGTACAaatccttcaga
	EU032179	gtattaaatgaaGGAACAAGTGGAACAGCTGTTACAACTAGTACACCTGGTTCAAAGGGTTCAGGTGGCTCAGTTGCTTCAGGTGGCTCAGTTGCTTCAGGTGGCTCAGTTGCTTCAGGTGGCTCAGTTGCTTCAGGTGGTTCAGTTGCTTCAGGTGGCTCAGTTGCTTCAGGTGGTTCAGTTGCTTCAGGTGGTTCAGGTGGCTCAGTTGCTTCAGGTGGTTCAGGTAATTCAAGACGTACAaatccttcagat
	M77714	gtattaaatgaaGGAACAAGTGGAACAGCTGTTACAACTAGTACACCTGGTTCAGGTGGTTCAGTTACTTCAGGTGGTTCAGTTACTTCAGGTGGTTCAGTTACTTCAGTTGCTTCAGTTGCTTCAGTTGCTTCAGTTGCTTCAGTTGCTTCAGGTGGTTCAGGTAATTCAAGACGTACAaatccttcagat
	M77721	gtattaaatgaaGGAACAAGCGGAACAGCTGTTACAACTAGTACACCTGGTTCAGGTGGTTCAGTTACTTCAGGTGGTTCAGTTACTTCAGGTGGTTCAGGTGGTTCAGTTGCTTCAGTTGCTTCAGTTGCTTCAGTTGCTTCAGGTGGTTCAGGTAATTCAAGACGTACAaatccttcagat
	HM153255	tattaaatgaaGGAACAAGTGGAACAGCTGTTACAACTAGTACACCTGGTTCAGGTGGTTCAGTTACTTCAGGTGGTTCAGGTAATTCAAGACGTACAaatccttcagat

RO-33	M32114	aaaatggtattaAAGGATGGAGCAAATACTCAAGTTGTTGCAAAGCCAGTACCTGCTGTAAGTACTCAAAGTGCTAAAAATCCTCCAGGTGCTACAGTACCTTCAG GTACTGCAAGTACTAAAGGTGCTATAAGATCTCCAGGTGCTGCAaatccttcagat
	DQ485448	aaaatggtattaAAGGATGGAGCAAATACTCAAGTTGTTGCAAAGCCTGCAGAAGCTGTAAGTACTCAAAGTGCTAAAAATCCTCCAGGTGCTACAGTACCTTCAG GTACTGCAAGTACTAAAGGTGCTATAAGTTCTCCAGGTGCTGCAaatccttcagat
	AB116598	aaaatggtattaAAGGATGGAGCAAATACTCAAGTTGTTGCAAAGCCTGCAGGTGCTGTAAGTACTCAAAGTGCTAAAAATCCTCCAGGTGCTACAGTACCTTCAG GTACTGCAAGTACTAAAGGTGCTATAAGATCTCCAGGTGCTGCAaatccttcagat
	AB715486	aaaatggtattaAAGGATGGAGCAAATACTCAAGTTGTTGCAAAGCCTGCAGGTGCTGTAAGTACTCAAAGTGCTAAAAATCCTCCAGGTGCTACAGTACCTTCAG GTACTGCAAGTACTAAAGGTGCTATAAGATCTCCAGGTGCTGCAaatccttcagat
	AB300614	aaaatggtattaAAGGATGGAGCAAATACTCAAGTTGTTGCAAAGCCTGCAGATGCTGTAAGTACTCAAAGTGCTAAAAATCCTCCAGGTGCTACAGTACCTTCAG GTACTGCAAGTACTAAAGGTGCTATAAGATCTCCAGGTGCTGCAaatccttcagat
