# Supplementary material for: An expanded global inventory of allelic variation in the most extremely polymorphic region of Plasmodium falciparum merozoite surface protein 1 provided by short read sequence data
Source: Malar J. 2018 Oct 1;17:345. doi: 10.1186/s12936-018-2475-2 (PMC6167803; doi:10.1186/s12936-018-2475-2)
Supplement: Supplementary file 5 — Additional file 5. The effect of k-mer length on the fraction of msp1 block 2 sequences assembled by Velvet. [file 12936_2018_2475_MOESM5_ESM.pdf]

Additional file 5.

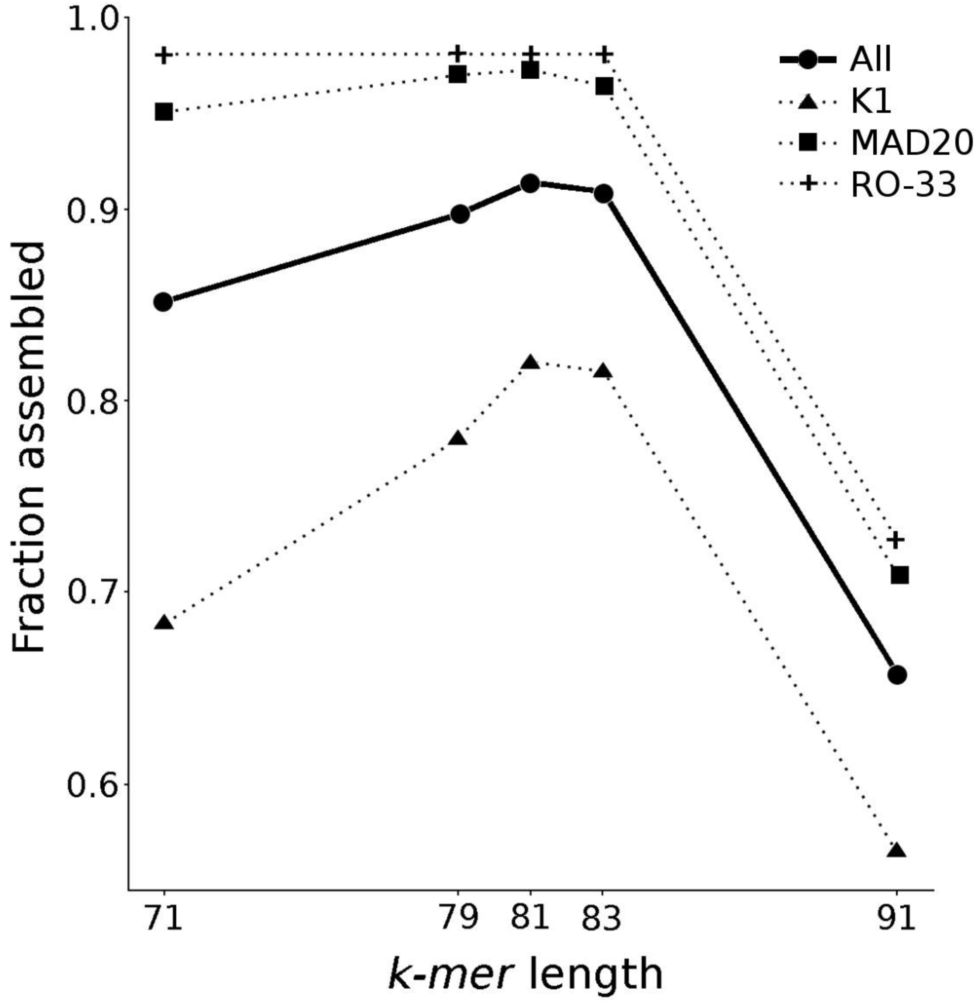

The effect of *k-mer* length on the fraction of msp1 block 2 sequences assembled by Velvet. 964 *msp1* block 2 sequences from previously published long reads were used to create dummy reads of 100 bp in length with a coverage of 50-fold. These reads were assembled using Velvet (Zerbino and Birney, *Genome Res* 2008, **18**:821-829) with a range of *k-mer* lengths. The resulting contigs were then scanned for the presence of the correct *msp1* block 2 sequence. The fraction of *msp1* block 2 sequences that were fully assembled for all 964 sequences is shown (solid line). The fraction of sequences fully assembled for *K1-like* sequences (triangles, n = 392), *MAD20-like* sequences (squares, n = 354) and *RO33-like* sequences (crosses, n = 204) are also shown (dotted lines).
