# Supplementary material for: An expanded global inventory of allelic variation in the most extremely polymorphic region of Plasmodium falciparum merozoite surface protein 1 provided by short read sequence data
Source: Malar J. 2018 Oct 1;17:345. doi: 10.1186/s12936-018-2475-2 (PMC6167803; doi:10.1186/s12936-018-2475-2)
Supplement: Supplementary file 6 — Additional file 6. Frequency distributions of length of msp1 block 2 sequence for assembled and unassembled sequences. [file 12936_2018_2475_MOESM6_ESM.pdf]

Additional file 6.

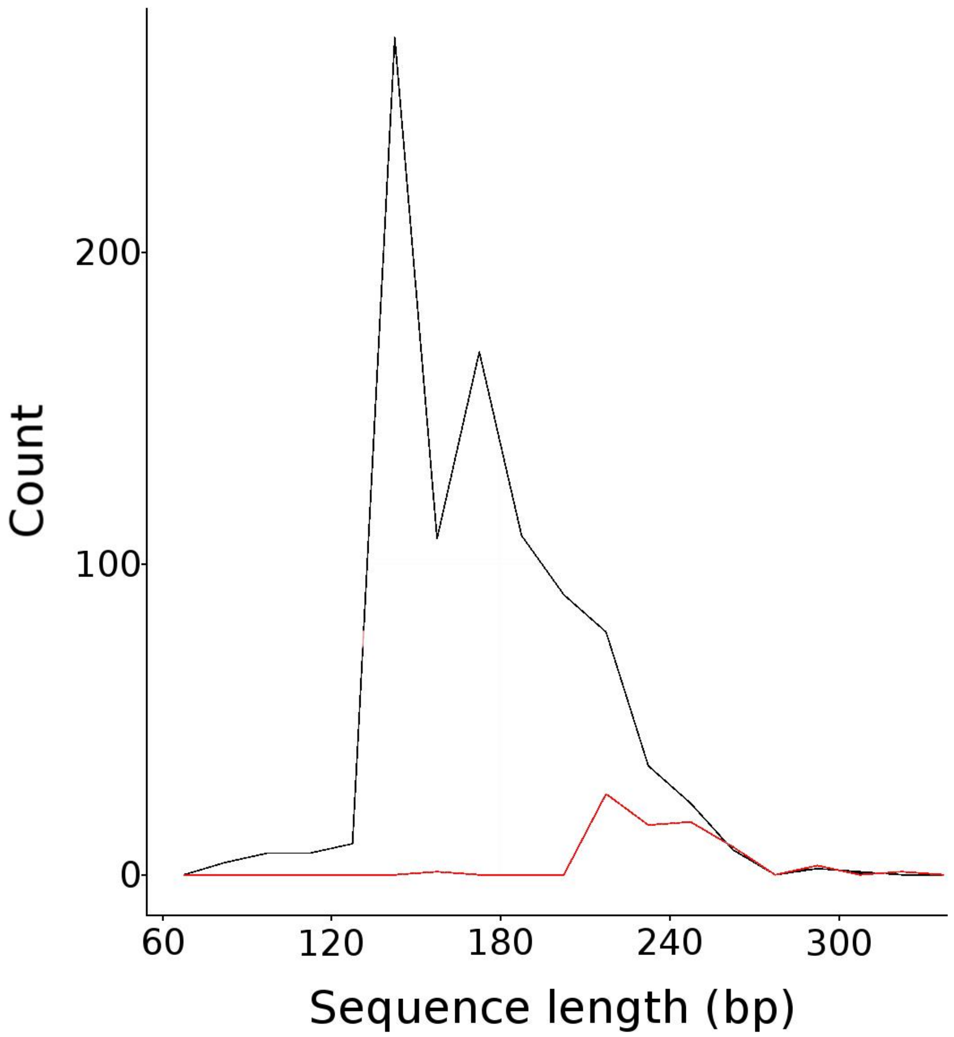

Frequency distributions of length of *msp1* block 2 sequence for assembled and unassembled sequences. Dummy reads created from 964 *msp1* block 2 sequences were assembled using Velvet. The frequency distributions of the lengths of the original *msp1* block 2 sequences are shown for successfully assembled (black line, n = 902) and unassembled (red line, n = 62) sequences are shown (sequences lengths are grouped with a bin width of 5 bp).
