# Supplementary material for: An expanded global inventory of allelic variation in the most extremely polymorphic region of Plasmodium falciparum merozoite surface protein 1 provided by short read sequence data
Source: Malar J. 2018 Oct 1;17:345. doi: 10.1186/s12936-018-2475-2 (PMC6167803; doi:10.1186/s12936-018-2475-2)
Supplement: Supplementary file 7 — Additional file 7. Probability of complete assembly of msp1 block 2 is dependent on depth of coverage. [file 12936_2018_2475_MOESM7_ESM.pdf]

Additional file 7.

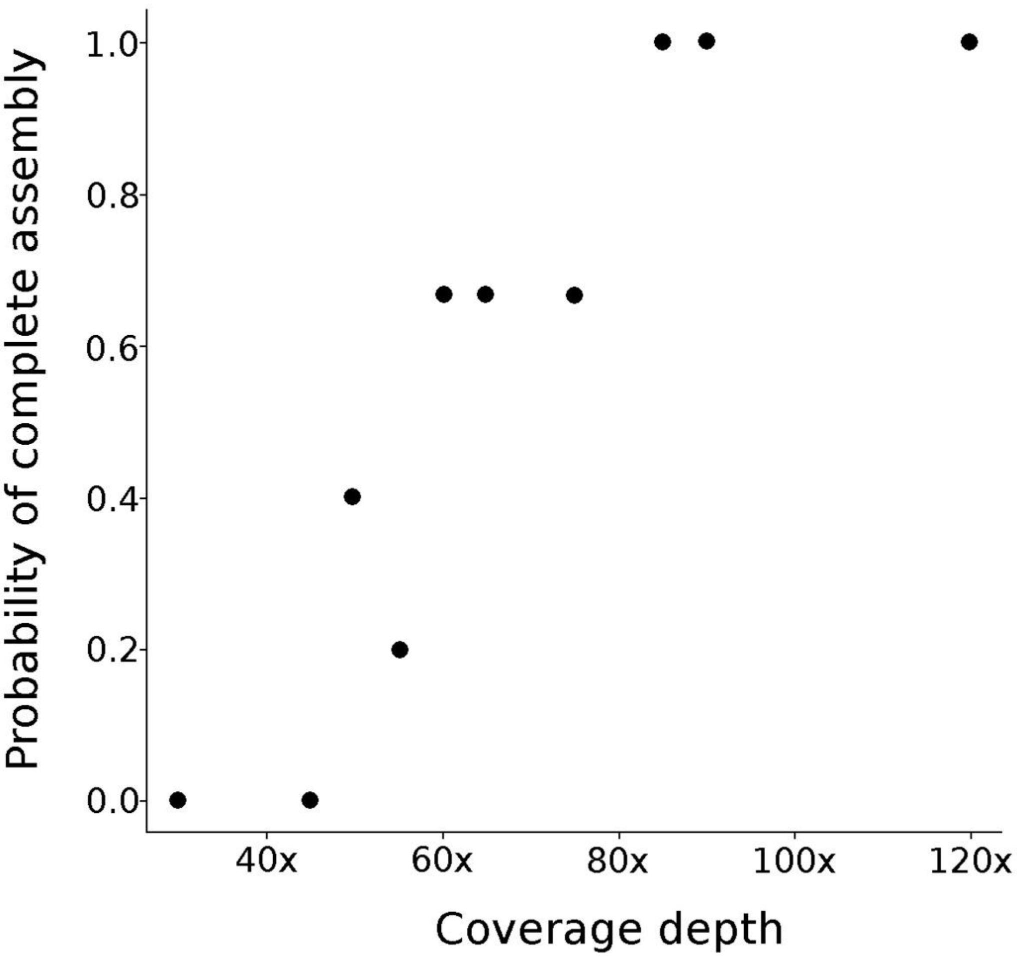

Probability of complete assembly of *msp1* block 2 is dependent on depth of coverage. Dummy reads were generated *in silico* from the Palo Alto allelic sequence of *msp1* block 2 at 10 different coverage depths. Reads were generated 10 times for each coverage depth and then assembled using Velvet, and the presence of the complete block 2 sequence was determined. The probability of assembling the whole *msp1* block 2 sequence is shown. There is a strong and significant correlation between coverage depth and the probability of complete assembly of the Palo Alto *msp1* block 2 sequence ($\rho = 0.96$, $p < 0.001$).
