# Supplementary material for: An expanded global inventory of allelic variation in the most extremely polymorphic region of Plasmodium falciparum merozoite surface protein 1 provided by short read sequence data
Source: Malar J. 2018 Oct 1;17:345. doi: 10.1186/s12936-018-2475-2 (PMC6167803; doi:10.1186/s12936-018-2475-2)
Supplement: Supplementary file 8 — Additional file 8. Distribution of coverage by allelic type after alignment of dummy reads to reference library. [file 12936_2018_2475_MOESM8_ESM.pdf]

Additional file 8.

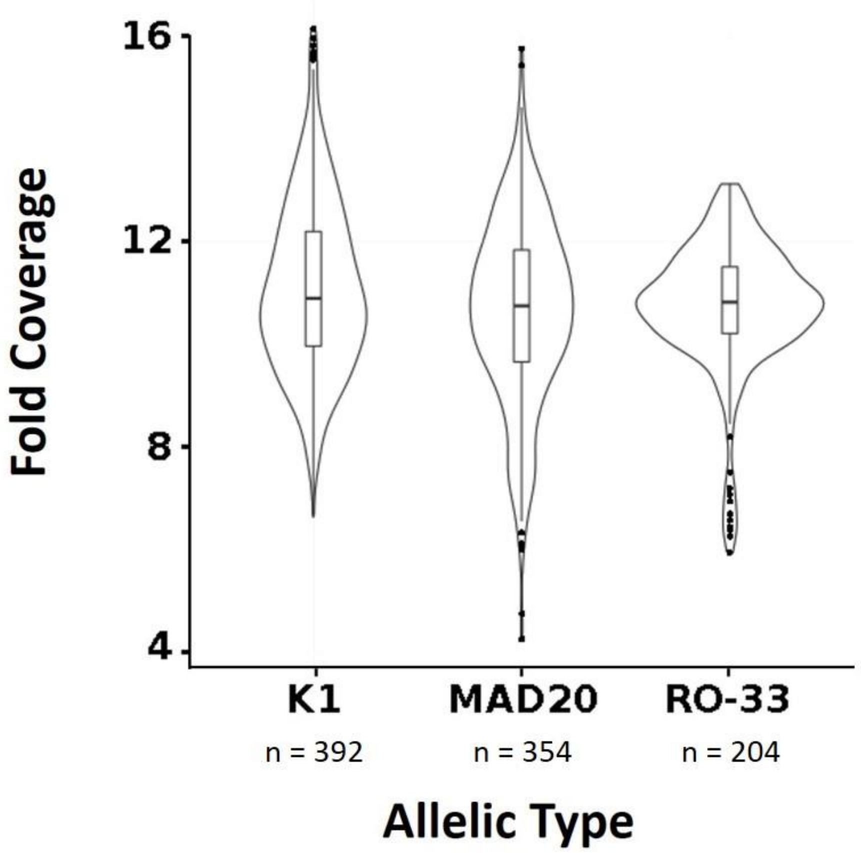

Distribution of coverage by allelic type after alignment of dummy reads to reference library. 100 bp dummy reads created from *msp1* block 2 long read sequences (Appendix files 1 and 2) were aligned to the reference library of 15 allelic sequences (Appendix file 4) using BWA-MEM. Coverage was calculated for each alignment and is shown for each major allelic type, showing no bias in the coverage among the types.
